# Orexins in the clinical course of bipolar disorder

**DOI:** 10.1192/j.eurpsy.2021.1657

**Published:** 2021-08-13

**Authors:** C. Moya-Lacasa, M. Valtueña-García, E. Martín Gil, L. González-Blanco, M.P. García-Portilla

**Affiliations:** 1 Psychiatry, SESPA Mental Health Services of Principado de Asturias, OVIEDO, Spain; 2 Department Of Psychiatry, University of Oviedo, Oviedo, Spain; 3 Neuroscience And Sense Organs, ISPA HEALTH RESEARCH INSTITUTE OF THE PRINCIPALITY OF ASTURIAS, Oviedo, Spain; 4 Psychiatry, CIBERSAM, Madrid, Spain

**Keywords:** bipolar disorder, Depression, orexins

## Abstract

**Introduction:**

Orexins are involved in the regulation of circadian rhythms which play an important role in mood regulation(1,2), and are hypothesised to be associated with major depressive disorder(3). However, scarce studies analyse their relationship with bipolar disorder (BD).

**Objectives:**

To evaluate the relationship of orexin-A and the clinical course of BD

**Methods:**

95 BD patients were tested for serum orexin-A. The clinical course was analysed through number of depressive, manic/mixed episodes. HDRS and YMRS were used to assess severity of current episode. Statistics: Spearman correlations, U Mann-Whitney, linear regression analysis.

**Results:**

Mean age was 50.03 (SD=12.87) and 64.2% were women. 63.2% had BD-type I. Mean number of manic, depressive and mixed episodes was 2.32 (SD=3.07), 7.28 (SD=12.37), and 3.01 (SD=9.06), respectively. Mean age of onset was 26.09 (SD=10.50). Mean concentration of orexin-A was 21.78 pg/ml (SD=15.41), with no differences in sex, body mass index, age at onset or presence of insomnia(ICD-10). A correlation with age was observed; r=0.24 (p=0.019). No association was identified between orexin-A and severity of current episode. In relation to clinical course, no correlation was found with manic or mixed episodes. However, a negative correlation was identified between orexin-A levels and number of depressive episodes; r=-0.36 (p=0.001). When linear regression (orexin-A as dependent variable) was used to control for age, only this covariate (B=0.304) entered in the model (R2=0.067, F=6.045, p=0.015).

**Conclusions:**

No relationship between orexin-A and number of manic/mixed episodes were detected. The association of orexin-A with number of depressive episodes dissappeared when age was controlled.

**Disclosure:**

No significant relationships.

